# Supplementary material for: A Humanized Yeast Phenomic Model of Deoxycytidine Kinase to Predict Genetic Buffering of Nucleoside Analog Cytotoxicity
Source: Genes (Basel). 2019 Sep 30;10(10):770. doi: 10.3390/genes10100770 (PMC6826991; doi:10.3390/genes10100770)
Supplement: Supplementary file 1 [file genes-10-00770-s001.zip › Additional_Files/Additional_File7_GOTermHeatmaps.zip!/Additional_File7_GOTermHeatmaps/exonucleolytic_nuclear-transcribed_mRNA_catabolic_process_involved_in_deadenylation-dependent_decay.pdf]

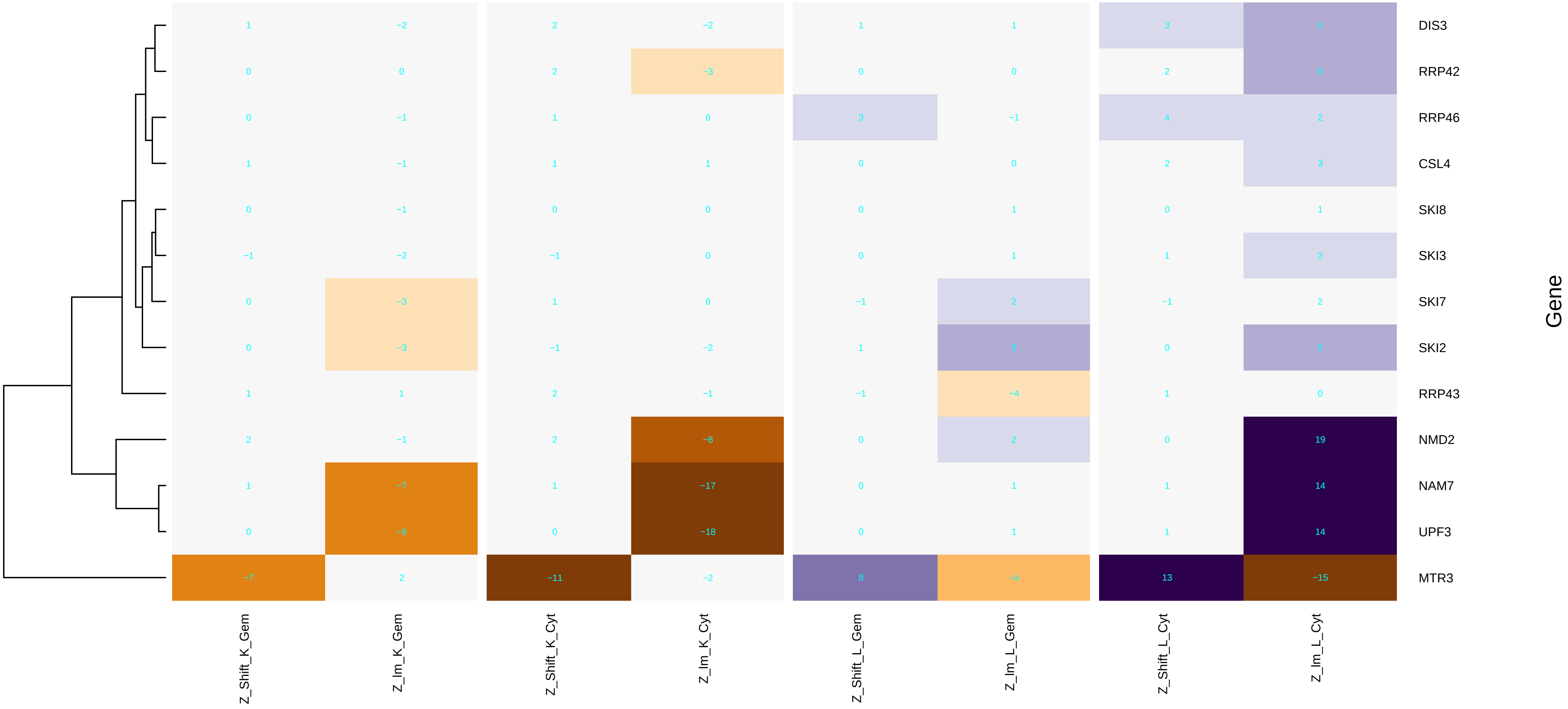

DIS3 1 −2 2 −2 1 1 3 5
RRP42 0 0 2 −3 0 0 2 6
RRP46 0 −1 1 0 3 −1 4 2
CSL4 1 −1 1 1 0 0 2 3
SKI8 0 −1 0 0 0 1 0 1
SKI3 −1 −2 −1 0 0 1 1 3
SKI7 0 −3 1 0 −1 2 −1 2
SKI2 0 −3 −1 −2 1 5 0 5
RRP43 1 1 2 −1 −1 −4 1 0
NMD2 2 −1 2 −8 0 2 0 19
NAM7 1 −7 1 −17 0 1 1 14
UPF3 0 −6 0 −18 0 1 1 14
MTR3 −7 2 −11 −2 8 −4 13 −15
Z_Shift_K_Gem
Z_Im_K_Gem
Z_Shift_K_Cyt
Z_Im_K_Cyt
Z_Shift_L_Gem
Z_Im_L_Gem
Z_Shift_L_Cyt
Z_Im_L_Cyt
Gene
